# One-Stop Shop: Diagnosis and Treatment of Basal Cell Carcinoma in One Step

**DOI:** 10.3390/jcm13133830

**Published:** 2024-06-29

**Authors:** Kristina Fünfer, Marco Mozaffari, Oliver Mayer, Sophia Schlingmann, Julia Welzel, Sandra Schuh

**Affiliations:** Department of Dermatology and Allergology, University Hospital, 86179 Augsburg, Germany

**Keywords:** one-stop shop method, basal cell carcinoma, margin marking

## Abstract

Monitoring the tumor margins of basal cell carcinomas is still a challenge in everyday clinical practice. Usually, the clinical margins of the tumor are marked by the naked eye or, even better, with dermoscopy before surgery and then examined in detail after the operation using histological examination. In order to achieve tumor freedom, several surgical steps are sometimes necessary, meaning that patients spend longer periods in hospital and the healthcare system is burdened more as a result. One way to improve this is the one-stop shop method, which requires precise diagnostics and margin marking before and during surgery so that tumor freedom can be achieved after just one surgery. For this reason, the current status of the diagnosis and treatment of basal cell carcinomas before and after surgery is to be examined following extensive literature research using devices and methods that have already been tested in order to determine how a simplified process of tumor margin control of basal cell carcinomas can be made possible both in vivo and ex vivo.

## 1. Introduction

The basal cell carcinoma (BCC) is still one of the most common types of skin cancer, with a steadily increasing incidence [1]. Because of various contributing factors, including UV radiation, the main locations of the lesions are sun-exposed regions of the face and the neck area [1]. We also see a growing world population with more elderly patients, showing the need for adapted treatment strategies to meet the growing demands [2].

Today, most patients undergo a biopsy when they have a lesion suspicious for BCC to confirm the diagnosis and plan the following treatment [3]. Some larger centers also use noninvasive imaging techniques for the skin, such as optical coherence tomography (OCT), reflectance confocal microscopy (RCM) or the latest development that combines both methods, the line-field confocal optical coherence tomography (LC-OCT). Once the diagnosis has been confirmed, the patient receives topical treatment or surgical removal, depending on the subtype and tumor thickness [4]. If surgery is required, the slow Mohs micrographic surgical technique is usually used in Germany for high-risk BCC. With this method, the patient sometimes has to undergo several operations, as a histopathologist has to search for tumor remnants in the margins after each removal, which takes up to 24 h. As a result, patients have a longer or additional stay in hospital, which causes higher costs for staff and materials and is stressful for the patients.

A standardized safety distance to the BCC is used for the margin mapping before excision, so it can be recognized that the procedure is not yet very individual [5]. Especially in the head area, where most BCCs are located, the skin must be treated carefully and sparingly to avoid unnecessarily large defects that may be difficult to close again [6]. For this reason, many colleagues believe that the invention of new noninvasive skin imaging technologies is needed to address these and future socio-economic challenges [7]. In order to ensure patient-centered and healthcare system-friendly therapy for BCC, the concept of the joint diagnosis and treatment of BCC in one procedure, the so-called BCC one-stop shop method should be pursued further. This article discusses which methods and devices are suitable for this concept.

## 2. One-Stop Shop

**Figure 1:**
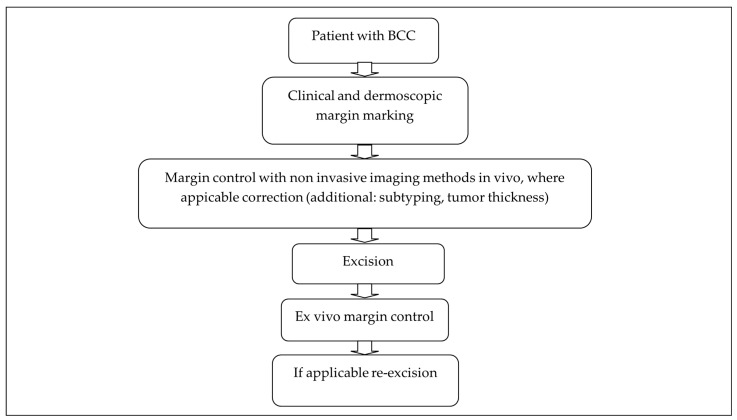
Management of a patient diagnosed with BCC using the OSS-M.

The idea of the one-stop shop concept (OSS-C) is the combination of the diagnosis and treatment of the patient in one day regardless of the method used for diagnostics or the type of therapy. For example, the patient comes to the consultation, the suspicious lesion is diagnosed with non-invasive imaging and then a therapeutic decision is made during the same appointment. This usually involves a prescription for topical therapy with, e.g., ointments, or a new appointment for photodynamic light therapy or excisions. This concept was already tested in one study for BCC where they diagnosed BCC with fresh-frozen biopsy and immediately performed surgery or local therapy (photodynamic therapy) [8]. The average throughput time of the 16 patients (with 19 tumors) included in this study was four hours and seven minutes [8]. All the patients were very satisfied with the concept, even if prior information was preferred for the patient’s personal planning [8]. In dermatology, there is also the idea of the one-stop shop method (OSS-M), which includes the subtyping and preoperative margin mapping of BCCs to avoid unnecessarily big surgery defects and re-excision in another surgical step. This method just addresses patients with already diagnosed BCC that are planned for a surgical excision (see Figure 1). To achieve this, there are certain factors that need to be considered and improved. These include a faster diagnosis of the BCC and its subtype, a more precise preoperative margin mapping to reduce revisions and a quicker review of the tumor remnants after excision. The various methods that can be used and that influence these factors are presented below.

### 2.1. Optical Coherence Tomography (OCT)

#### 2.1.1. Technical Details

OCT has been used in dermatology since 1997, particularly for non-melanocytic lesions [9]. The image is created by the different light absorption of the different skin components and can also show deeper structures, with a penetration depth of up to 2 mm [10]. The device emits a broad spectrum of near-infrared light that penetrates the skin. This light is split into two beams, with one directed at the skin and the other serving as a reference. When the light is reflected back from different skin structures, it interferes with the reference beam. A detector captures this interference pattern. A computer then processes the captured data to construct detailed cross-sectional images of the skin layers [11,12]. For example, a commonly used device, the VivoSight Dx^TM^ (Michelson Diagnostics Ltd., Maidstone, Kent, UK), scans with an image size of 6 mm × 6 mm [13]. It has a penetration depth of 1 mm into the dermis, with a resolution of <10 µm, using patented multi-beam technology (see Table 1 and Table 2) [13]. 

#### 2.1.2. Experiences with OCT

- In vivo

This procedure has already been shown to be an alternative to punch biopsy for the diagnosis of BCC, which can reduce unnecessarily invasive methods [69]. In this multicenter, randomized, non-inferiority study, 553 patients, aged 18 years or older, were evaluated. Patients who needed a biopsy on a lesion suspicious for a BCC located outside the H-zone (high-risk zone) of the face were randomly assigned to either receive OCT or a punch biopsy (standard care) through an online randomization system. A total of 95% of patients (250 out of 263) in the OCT group and 94% of patients (262 out of 278) in the regular care group were free from residual or recurrent lesions (premalignant or malignant). The absolute difference was 0.81% (95% CI −2.98 to 4.60; one-sided *p* = 0.34), proving that OCT-guided diagnosis and treatment of BCC is non-inferior to regular care punch biopsy [69]. 

In 2021, a consensus statement was published defining the characteristic features of BCC in OCT, such as hyporeflective areas, hyperreflective areas and ovoid structures, as well as other criteria for distinguishing the subtypes and differential diagnoses [25,70]. OCT-assisted diagnosis, laser therapy and treatment monitoring of BCC was also proven to be an effective treatment option [71].

There are also various studies that have investigated the use of OCT in preoperative margin mapping. In a case report, it could be demonstrated that the defect in Mohs surgery was comparable to the preoperative OCT-marked margins using previously taken photos [26]. In another study, the preliminary dermatoscopic margins were adjusted in four out of ten patients by adding an additional 2 mm safety margin when the tumor was still present in the OCT picture, leading to eight out of ten completely excised BCCs in a single step [72]. A recently published single-center study also showed good agreement between OCT and histology by preoperatively examining the margins of biopsy-proven BCC for tumor remnants and then verifying these results with the gold standard histology [27]. For 22 patients with BCC, OCT was used to map the lesion borders before Mohs micrographic surgery. Histology showed no BCC in 7 out of 22 cases (32%), correctly analyzed by OCT in 6 cases (86%). Of the nine tumors (41%) requiring a single MMS stage, OCT accurately predicted this in seven cases (78%). For the six tumors (27%) requiring two MMS stages, OCT determined the need for a second stage in five cases (83.3%). Overall, OCT diagnosed BCC with 95.5% accuracy (κ = 0.89, *p* < 0.01) [27].

- Ex vivo

It is possible to analyze samples ex vivo with OCT. In a study by Cunha et al., 75 freshly excised sections of 38 BCC were imaged with this device and compared with fresh-frozen histology, but the tumors were difficult to visualize [28]. Only in 4 out of 26 positive hematoxylin–eosin cuts and 23 out of 49 negative sections did the OCT images show good correlation. The sensitivity was 19% and the specificity was 56% [28].

### 2.2. Reflectance Confocal Microscopy (RCM)

#### 2.2.1. Technical Details

Reflectance confocal microscopy (RCM) is a non-invasive imaging technique used in dermatology to visualize the skin in real time, especially melanocytic lesions [73]. It operates by using a low-power laser, typically in the near-infrared range, to penetrate the skin. The reflected light from the laser comes back from different layers of the skin with various intensities due to the cellular structures and the differences in the refractive index within the skin. The good contrast and resolution of the image is secured by a pinhole aperture filtering out the out-of-focus lights. The reflected light is detected and converted into a digital signal to form a complete grayscale image. By adjusting the focal plane of the laser, images can be captured at various depths, allowing for the creation of a detailed, three-dimensional representation of the skin’s structure. It also allows dermatologists to monitor the effectiveness of treatments for skin conditions without the need for repeated biopsies [74,75,76].

For this technique, there are different devices for in vivo or ex vivo use.

For in vivo, either the VivaScope^®^ 1500 (VivaScope GmbH, Munich, Germany) or the VivaScope^®^ 3000 (VivaScope GmbH, Munich, Germany) are usually used, which are available individually or in combination from the manufacturer. As the name of the technology suggests, the device works with a laser (830 nm) to visualize various skin structures. Both devices have a resolution of <1.25 µm in the horizontal image and <5 µm in the vertical mode. While the VivaScope^®^ 1500 can only measure a maximum size of 8 mm × 8 mm in 0.5 mm × 0.5 mm steps, the VivaScope^®^ 3000 handheld device can explore an unlimited size 0.75 mm × 0.75 mm steps (see Table 1) [14]. 

The ex vivo device is the VivaScope^®^ 2500M-G4 (VivaScope GmbH, Munich, Germany), which works with two different lasers, one with the wavelength of 488 nm and the other with 785 nm. It shows, after dying the tissue with fluorescence dye, the structures on a cell level. The other laser generates a reflectance signal. Together with these two pieces of information, the algorithm can create a picture that looks like the histopathological H&E coloring. It has a great optical resolution, with <1.25 µm horizontal and <5.0 µm vertical, with a maximum sample size of 25 mm × 25 mm and a penetration depth up to 200 µm. The possible magnification for the picture taken is 550× (see Table 1 and Table 2) [15].

#### 2.2.2. Experiences with RCM

- In vivo

RCM is a well-studied method for assessing skin lesions in dermatology. It has already been shown several times that the diagnostic accuracy and monitoring of BCC with an additional RCM examination is better than with dermoscopy alone [29]. Kadouch et al. showed in their two-centered study with 100 BCC patients that the chance of detecting a BCC with an in vivo RCM procedure is comparable to punch biopsy and subsequent histology (sensitivity 100% RCM vs. 93.94% punch biopsy, specificity RCM 38% vs. 79% punch biopsy), although the classification into subtypes is poorer with RCM (e.g., for infiltrative BCC subtypes 50% to 85% with RCM vs. 77% by punch biopsy) [30].

RCM has also been tested by Navarrete-Dechent et al. for its ability to reveal tumor remnants of BCC after biopsy in 61 patients without clinically visible residuals, which could likely avoid unnecessary surgery [77]. Some 73.8% of patients showed BCC residuals with RCM, and 68.9% in histology. The sensitivity and specificity of RCM were 92.8%, and 68.4%. Three high-risk, infiltrative, and basosquamous BCCs were missed using RCM [77].

Attempts were also made to use in vivo RCM for margin mapping. To verify that the RCM margin reflects the actual tumor margin, Pan et al. compared the results with a frozen biopsy and showed good agreement with the lateral margins but difficulty with the depth of the tumor, meaning that histologic examination was still required [31]. In 12 out of 13 cases (92.3%), frozen biopsy confirmed that the surgical margins delineated by RCM were clear [31]. One consideration, reviewed by many others, was to confirm the margins initially created with dermoscopy with an RCM in vivo examination to better detect subclinical remnants [32,33]. Venturini et al. detected BCC residuals beyond the dermoscopic margin in 3 out of 10 lesions confirmed by histology [32]. Even inexperienced users could delineate the margins of 17 BCC patients better with a handheld RCM (median RCM-predicted and observed surgical defect areas (2.95 cm^2^ [range: 0.83–17.52] versus 2.52 cm^2^ [range 0.71–14.42]; *p* = 0.586) than with dermoscopy alone (median area of 1.34 cm^2^ [0.41–4.64] versus 2.52 cm^2^ [range 0.71–14.42]; *p* < 0.001) [34]. This was demonstrated by comparing images of the RCM-predicted margins with images of the surgery defect, where dermoscopy often underestimated the real tumor [34].

To combine the use of OCT and RCM, a device was invented that allows better diagnosis of BCC and BCC remnants as well as visualization of the deep and lateral margins, and to prove this, Mohs defect images were compared with these measures [35]. RCM–OCT showed an overall agreement of 91.1% with MMS frozen sections. The depth measurements from OCT were highly correlated with those from MMS, with an r^2^ value of 0.9 [35].

The RCM procedure has already been tested in a one-stop shop concept where the diagnosis of a BCC and its subtype was confirmed using RCM [36]. Afterwards, the BCC was excised with a predefined safety margin, depending on the subtype, and finally, compared with the standard concept of punch biopsy for diagnosis and subsequent surgery [36]. In this open-label, parallel-group, noninferiority, randomized controlled multicenter study, 40 out of 40 patients in the one-stop shop group were free of BCC residuals, whereas in the standard-care group, 2 out of 33 patients did not show tumor free margins. Therefore, noninferiority was proven [36].

Another potential application that has already been tested is the intraoperative in vivo application of RCM to shave biopsy wounds previously prepared with aluminum chloride (AlCl_3_) to increase the contrast to see the tumor remnants directly [37].

- Ex vivo

As mentioned above, the RCM device can be used in different modes, such as reflectance mode, fluorescence mode or a combination of both modes, for the detection of BCC and ex vivo monitoring of tumor margins. Based on three simple criteria, ex vivo diagnosis of BCC should be possible for both trained and inexperienced RCM users alike [38]. This was demonstrated by the fact that confocal experts correctly diagnosed 110 out of 116 BCCs and novices 107 out of 116 BCCs [38]. However, another study in which dermatohistopathologists with different levels of experience were asked to assess BCCs in RCM images concluded that experience with this device plays an important role in detecting tumor remnants [39]. The two inexperienced examiners achieved sensitivities of 59.5% and 71.1% and specificities of 94.8% and 89.8%. The experienced reader achieved a sensitivity of 78.5% and a specificity of 84.8% [39]. The automated deep-learning algorithm developed by Sendín-Martín et al. could help with this problem, as it is designed to assist the user with the diagnosis of BCC [78]. In addition, Kose et al. showed in an international three-center study that a training program for the interpretation of BCC in fluorescence mode could help to increase the confidence of the diagnosis [79]. The readers achieved an average sensitivity of 90% and a specificity of 78% in detecting residual BCC margins across 30 samples [79]. Also, to ensure an easier diagnosis, especially for histology experts, the fusion of both modes by staining the skin with acridine orange and acetic acid produces a hematoxylin–eosin-like image that is easier to interpret [40]. It also does not interfere with subsequent histology [40].

Fresh tissue should be used for the RCM examination, even though frozen tissue is possible in principle, but it causes more artefacts [80].

### 2.3. Line-Field Optical Coherence Tomography (LC-OCT)

#### 2.3.1. Technical Details

Unlike traditional confocal microscopy, which uses a point source of light, line-field confocal microscopy (LC-OCT) employs a line-shaped light source to illuminate the skin. This line of light scans across the tissue, illuminating a thin strip of skin at a time. The reflected light from this line illumination is captured by a detector. A confocal pinhole filters out-of-focus light, ensuring that only the light from the focal plane contributes to the image. This enhances the contrast and resolution, allowing for detailed visualization of cellular and subcellular structures within the skin. LC-OCT can rapidly scan large areas of skin by moving the line of light across the surface, creating high-resolution images much faster than point-scanning methods [81,82].

So, LC-OCT combines the principles of RCM and OCT. The deepLive^TM^ device (DAMAE Medical, Paris, France) has a high resolution and three different modes for visualizing the various skin structures. A vertical mode with an image size of 1.2 mm × 0.4 mm, a horizontal mode with an image size of 1.2 mm × 0.5 mm and the creation of 3D blocks with the size of 1.2 × 0.5 × 0.5 mm^3^ are possible. Live skin scanning is possible for both 2D modes thanks to the fast image acquisition of 8 frames per second. A resolution of up to 1.3 µm is also possible. In order to obtain a good image quality, a drop of paraffin oil must be applied to the patient’s skin to serve as a medium. A simultaneous dermatoscopic image of the skin on the lower right screen is taken with the same device and helps with the orientation on the patient (see Table 1 and Table 2) [16].

#### 2.3.2. Experiences with LC-OCT

- In vivo

As with the other devices, LC-OCT can be used to visualize various skin tumors, but with high resolution [41]. Due to the excellent image quality, some specific criteria for BCC and its subtypes have also been established [83]. Suppa et al. analyzed 89 BCCs and detected frequent criteria for BCC, like lobules, blood vessels, and small bright cells in the epidermis/lobules. The LC-OCT criteria for superficial BCC were hemispheric lobules, no separation from the epidermis, and no stromal stretching; for nodular BCC, bigger lobules and no connection to the epidermis; and for infiltrative BCC, branched lobules [83]. These criteria were used in a multicenter study on 182 lesions of 154 patients to demonstrate that LC-OCT can confirm the diagnosis of BCC in dermoscopically unclear lesions, thus increasing the sensitivity (98%) compared to dermoscopy alone (90%) [42]. One article also showed a high agreement of over 90% in the determination of subtypes with LC-OCT compared to the gold standard, histopathology [43]. Thus, the literature indicates that BCC is the most suitable skin tumor to be examined with the LC-OCT [84].

This non-invasive imaging has also been examined in a pilot study, which showed that the use of this method can also be useful in the follow-up of topically treated BCCs [85]. In this case, 20 superficial BCCs from 12 patients treated with imiquimod 5% cream 5 days a week for 6 weeks were examined [85]. Subclinical remnants were found in three patients 4 weeks after the end of the treatment despite complete clinical response [85].

Recently, Jacobson et al. published a case study in which LC-OCT was used for the preoperative planning of an infiltrative BCC to save skin and possible additional surgical steps [44]. It was also recently shown in a case-control study of 63 facial high-risk BCCs from 60 patients that the Mohs steps (LC-OCT group of 22 BCCs: 1.23 ± 0.43 SD, and control group of 41 BCCs: 1.89 ± 1.05 SD, respectively; *p* = 0.007) can be reduced if the margins of high-risk BCCs are evaluated beforehand using LC-OCT [45]. Moreover, LC-OCT imaging showed near-perfect agreement with histopathology in distinguishing the low- and high-risk recurrence BCC subtypes (Cohen’s kappa 0.88) [45].

**Figure 2:**
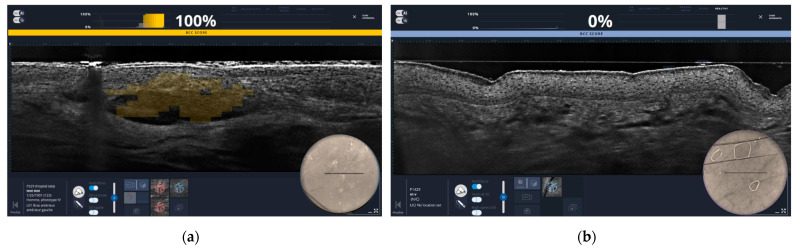
These are two images from the LC-OCT software with explainable artificial intelligence (XAI). Six score categories can be differentiated: actinic keratosis, squamous cell carcinoma, melanocytic lesions, sebaceous hyperplasia, dermal nevus, other and healthy skin. (**a**) The tumor nodules of a basal cell carcinoma (BCC), highlighted as a yellow heatmap and with a BCC score of 100%, which indicates the probability of the presence of a BCC. (**b**) The regular architecture of healthy skin. In this image, no heatmap is shown and the BCC score is 0%. There is therefore no probability that a BCC is present in this image.

LC-OCT also has integrated explainable artificial intelligence (XAI) for the detection of BCC. The prototype of the XAI marks the tumor nodes of the BCC as heat maps overlaid on the LC-OCT images in real time during the measurement. There is one main BCC score between 0 and 100%, which is associated with a color from blue to yellow. The yellow color corresponds to a probability of the presence of BCC in the displayed area of 50% or more, while the blue color indicates a probability of less than 30% (Figure 2).

- Ex vivo

Currently, there are attempts to combine LC-OCT with confocal Raman microspectroscopy in order to determine the molecular characteristics of ex vivo skin material [86].

### 2.4. High Frequency Ultrasound (HFUS)

#### 2.4.1. Technical Details

High-frequency ultrasound in dermatology uses sound waves with frequencies typically above 20–50 MHz to create detailed images of the skin. These sound waves penetrate the skin and reflect off different tissues. The reflected waves are captured to form images, allowing visualization of skin layers and structures. This technique helps in diagnosing skin conditions, assessing tumor depth, and monitoring treatment effects [87].

With this further development of ultrasound and the use of high-resolution transducers above 10 MHz, this technique has also become an option for dermatological imaging [17]. A distinction is also made between high-frequency ultrasound with transducers between 20 and 30 MHz and transducers with over 30 MHz, which are counted as ultra-high-frequency ultrasound [17]. Thanks to this high resolution, the first skin layers down to the deep fascia can now be better assessed (see Table 1 and Table 2) [17]. The HFUS can also be used as an ex vivo device, as you can scan the tissue with ultrasound gel like it was in vivo, which Pasquali et al. already tested in their study (see Table 1 and Table 2) [46].

#### 2.4.2. Experiences with HFUS

- In vivo

This technique categorizes BCC more easily into the high-risk or low-risk subtypes than the macroscopic and dermatoscopic assessment tested in this study, which described several criteria found in the high-risk subtypes [47]. One study tested three different transducers (13 MHz, 20 MHz, 40 MHz) on non-melanoma tumors of the head and neck area [48]. Tamas et al. found that the 13 MHz transducer was best for imaging surgical margins and large lesions, but at the expense of the lower quality for a larger image [48]. Consequently, for more detailed examinations, for example, to show the hyperechoic spots typical of BCC, the 20 and 40 MHz transducers were more suitable [48]. A water-based correction fluid was tested for drawing the margins on the skin when using HFUS [88].

Although there is not much literature on HFUS in the margin mapping of BCC, Janowska et al. see great potential in this technique to improve the surgery [49].

- Ex vivo

Compared to histology, this technique shows good results in the detection of tumor remnants at the margins in a prospective, single-blinded study [46]. A total of 84 malignant lesions (each with a maximum length of 13 mm and maximum depth of 8 mm) of 100 tumors in 89 patients were evaluated. A total of 79 of the 84 malignancies were BCCs. Moreover, 81 out of 84 malignant lesions correlated between ex vivo HFUS and histology with 77 negative and 4 positive borders [46].

### 2.5. Dermoscopy

#### 2.5.1. Technical Details

Dermoscopy is a technique that has been known and used in dermatology for some time. In principle, the lesion in question should be magnified and illuminated by the dermatoscope. The magnification ranges from ×10 to ×200 and can therefore show skin structures up to the reticular dermis [18]. The light source in the dermatoscope can be either polarized or non-polarized. Polarized light reduces the surface reflection, providing a clearer view of deeper skin structures, while non-polarized light enhances the visualization of surface features. Some dermatoscopes can switch between both types of light, offering flexibility based on the examination needs. To enhance the clarity of the image and to reduce the light scattering from the skin surface, a liquid interface, such as alcohol, oil, or gel, is often applied between the skin and the lens. The technical function of dermoscopy relies on the principle of transillumination of deeper structures, allowing the dermatologist to observe intricate details of the lesion (pigment patterns, vascular structures, architecture of the skin layers) [89,90].

As there are now many different manufacturers of dermatoscopes, they differ in their additional features, such as a built-in camera for taking photos or core polarizers for better visualization of the structures (see Table 1 and Table 2) [18].

#### 2.5.2. Experiences with Dermoscopy

- In vivo

Dermoscopy is probably the most frequently used tool in dermatology for a closer examination of the skin. There are also specific criteria for the diagnosis and classification of subtypes of BCC [91]. Some typical dermatoscopic findings for BCC are: blue–gray globules, ulcerations, maple leaf-like structures, blue ovoid nests, arborizing vessels and some more [92]. Classification into subtypes is more difficult according to one study, as they tried to find specific structures for each subtype in 102 BCCs [50]. Typical criteria like arborizing vessels, especially in aggressive BCC, were sometimes hard to detect, while superficial BCCs are easier to diagnose because of the short, fine telangiectasias. Subsequently, nodular BCCs were also well identified due to the blue–gray ovoid nests [50]. In addition to this limitation, a study with 934 BCCs from Japan points out that the sensitivity for non-pigmented BCCs (76.5% (CI 59.8–87.8) is worse than for pigmented BCCs (between 93.2% (CI 90.3–95.3) with a pigmentation between 10–50% and 95.4% (92.5–97.3) for BCC with a pigmentation of >50%) [51]. Due to this fact, they might miss a diagnosis with dermoscopy [51]. However, the average size of a pigmented BCC at initial diagnosis has decreased with the introduction of dermoscopy in clinical practice (median tumor size, 10.0 mm before dermoscopy, 8.0 mm after introduction of dermoscopy; Mann–Whitney U-test, *p* = 0.011) [93]. This could mean that BCCs are detected earlier [93]. The fact that the specificity and sensitivity for pigmented BCCs are higher than for non-pigmented BCCs was also demonstrated in a systematic review [94]. They also looked at the diagnostic accuracy of dermoscopy (85% sensitivity and 98.2% specificity) compared to the naked eye (66.9% sensitivity and 97.2% specificity) [94]. Reiter et al. and concluded that dermoscopy is better, especially when performed by experts directly on the patient and not just diagnosed based on dermoscopic images [94]. It was expected that this tool could also improve margin marking for surgery. However, it has often been shown that there is no significant difference in the number of surgical steps. This issue was demonstrated in a study of 60 patients with BCCs in the head and neck area, which were randomized in three groups comparing the naked eye, curettage and dermoscopy (*p* = 0.1) [52]. Gurgen et al. also found no difference in the number of stages when specifically marking 40 infiltrative BCCs with the naked eye and dermoscopy [53]. In the single-center study by Chen et al., with 107 BCC patients, dermoscopy was also compared with visual margin marking of BCC, and a discrepancy of 16.8% was found between the two markings [54]. Subsequently, the BCCs were removed with an additional safety margin of 5 mm from the dermoscopic margin and examined histologically afterwards [54]. It was shown that tumor freedom could be found in 98.1% with 4 mm as an additional safety distance to the dermoscopic margin in all the BCCs and 92.3% with 2 mm in the pigmented BCCs [54]. Caresana et al. also found in their study of 200 BCCs on the head and neck area that dermoscopy allowed the better detection of tumor remnants in 69 cases (34.5%) that would have been missed by the naked eye [55]. Another systematic review on the use of dermoscopy for margin delineation in Mohs surgery confirms that dermoscopy has the same number of Mohs surgical steps compared with visual marking, but the lateral margins are less frequently the cause of re-intervention [56]. This is leading us to believe that dermoscopic margin delineation is superior to that by the naked eye [56]. There is a study that aimed to use a smaller safety distance in pigmented BCC due to the use of dermoscopy [57]. Ito et al. showed in their study of 288 pigmented BCCs, in which 218 (75.7%) were excised with a smaller border (≤3 mm) and 60 (24.3%) with a larger one (≥4 mm), that 99% of the cases with a narrow safety distance were completely excised [57].

- Ex vivo

For ex vivo dermoscopy, there is not much literature. Only one study recommends the use of ex vivo dermoscopy with derm-dotting for pathologists who examine skin biopsies to achieve a more targeted approach in diagnosing [95]. The detection of positive section margins in non-melanoma skin cancer increased from 8.4% to 12.8%, especially for superficial BCC [95]. Derm-dotting is the marking with nail polish of suspicious areas seen in dermoscopy, for example, in order to correlate them better later in histology [96].

### 2.6. Other Tools

#### 2.6.1. Hyperspectral Imaging System (HIS)

Hyperspectral imaging systems (HISs) capture detailed images across a wide range of wavelengths. These systems collect spectral data from the skin, producing images that reveal subtle variations in the tissue composition [97]. The HIS generates hyperspectral graphs for each pixel, which a computer can use to create a localization map of the tumor; for example, to delineate the BCC margins [97]. This was tested by Salmivuori et al. and subsequently compared with histology and clinical marking [97]. In this pilot study, the borders of 12 out of 16 BCCs were better marked by HIS in comparison to the clinical assessment (4 of 16 had wider margins and 8 of 16 smaller ones by HIS). In 2 out of 16 cases, HIS missed the subclinical BCC extension, and in 2 out of 16 cases, HIS estimated a larger BCC expansion [97]. There is also a study that concluded that some BCCs were more difficult to delineate [98].

#### 2.6.2. Polarization-Enhanced Reflectance and Fluorescence Imaging (PERFI)

Polarization-enhanced reflectance and fluorescence imaging (PERFI) uses polarized light to improve the visualization of skin tissues. This technique captures good-contrast images by analyzing the way polarized light is reflected or emitted by the skin by filtering out scattered light. This method also detects fluorescence from skin tissues, revealing additional information about their molecular properties [99]. In this method, methylene blue is injected into the area of the non-melanoma skin cancer in eight patients in combination with anesthetic solution, which resulted in the blue contrasting of the tumor and enabled good in vivo delineation of the tumor. The correlation of the ex vivo images with the histology was good [99].

#### 2.6.3. Rapid Lump Examination (RLE)

Rapid lump examination (RLE) is the rapid microscopy of a surgical specimen’s (lump) surface and subsurface structures. It provides immediate feedback on the lump’s size, shape, and internal composition [100]. In this study, the freshly excised tissue is stained and examined directly with a digital microscope or a stereo microscope in order to be subsequently compared with the histological results. The staining has no influence on the subsequent histology. Both microscopes performed well, with a 91% sensitivity and 90% specificity for the 129 specimens examined using a digital microscope and 90% sensitivity and 94% specificity for the 78 samples using a stereo microscope [100]. Moehrle et al. also developed an immunohistological protocol with the antibody BerEP-4 [100]. In the study by Peters et al., 382 specimens out of 118 suspected BCCs showed a sensitivity of 76% and a specificity of 91% [101].

#### 2.6.4. Confocal Laser Endomicroscopy (CLE)

Confocal laser endomicroscopy is a confocal microscope at the end of a coloscopy tube, which enables direct in vivo histology in gastroenterology [102]. This method was also tested in dermatology. It involves a tiny laser and a detector to capture detailed images. CLE employs confocal microscopy principles to show cellular structures and abnormalities of the skin tissues in vivo [103,104]. It is superior to magnifying glasses in outlining the tumor margins before surgery in BCCs and squamous cell carcinomas in a prospective study of 19 patients. However, 27% of the CLE group still had to be re-excised [103].

#### 2.6.5. Fluorescence Diagnosis (FD)

Fluorescence diagnosis (FD) uses special light to make certain skin cells and tissues emit fluorescence. A photosensitizing agent is often applied to the skin, which accumulates in abnormal cells. When exposed to a specific wavelength of light, these cells fluoresce, emitting light that is captured by a camera [105]. Jeon et al. stained 258 BCCs of 255 patients in their study in vivo with a photosensitizer (here: 20% aminolaevulinic acid) to make the extensions of the BCC visible under the Wood light and subsequently to better mark the margins. This technique was tested in 199 pigmented and 59 non-pigmented BCCs, whereby FD was only able to reduce the steps of the Mohs surgery in non-pigmented BCC [105].

## 3. Discussion

As described above, two basic types of one-stop shop can be distinguished, each of which can also benefit from the use of different devices. On the one hand, we have the general OSS concept (OSS-C), which is intended to enable diagnosis and direct surgical or topical treatment decisions in one session. This concept has already been tested in a study for basal cell carcinoma (BCC), with fresh frozen sections for diagnosis and photodynamic therapy or excision on the same day [8]. On the other hand, it is a specific OSS method (OSS-M) that requires precise margin marking with classification of the BCC subtype and subsequent fast scanning of the margins before excision to perform surgical therapy in one step.

With the OSS-C, it is important to have a device that reliably detects BCC and can perform a good subtyping in order to make the subsequent treatment decision. A study has already looked at this concept and found that with certain changes, the throughput times of a patient can be reduced by 90% with the same resources, causing a positive effect on the patient through rapid diagnosis and treatment [106]. The dermatoscope does not seem to be well suited for this, as it cannot reliably subtype the BCC, especially when it comes to aggressive BCCs [50]. High-frequency ultrasound seems to be superior, as it can differentiate well between the low- and high-risk types of BCC, which is important for treatment decisions [47]. The subtyping of BCC with line-field confocal optical coherence tomography (LC-OCT) agrees with the histology in over 90% of cases and could therefore be the appropriate device [43]. Reflectance confocal microscopy (RCM) is poor at subtype classification in vivo compared to histology [30], although a study has already tested an OSS-C with RCM and achieved good results [36]. Moreover, optical coherence tomography (OCT) can detect the subtype moderately, also highly depending on the image quality, which can be affected by scales and crusts [66]. Nevertheless, it needs to be considered that with the OSS-C some patients may not be prepared for immediate treatment after diagnosis; for example, patients need to sign the informed consent 24 h before undergoing a surgical procedure, some medication needs to be discontinued beforehand or the individual risk must be assessed preoperatively. This is the reason why the OSS-C works well for therapies that can be carried out at home or for planning therapeutic options (like photodynamic therapies or surgeries), but for excisions on the same day a lot of logistics need to be considered.

For diagnosis and excision on the same day (the OSS-C), only specific patient categories would be suitable candidates for this approach. Small lesions in easily accessible areas, e.g., on the trunk, are particularly suitable for this in younger patients who do not need to be taken off anticoagulation medication beforehand and for whom prior written consent could be obtained, e.g., by means of teledermatology. These are just suggestions that have to be proven in further studies.

The OSS-M integrates subtyping, preoperative and postoperative margin control of BCCs into a single session, aiming to streamline the treatment process, minimize unnecessary surgery defects, and reduce the need for re-excisions. Patients will benefit from fewer surgical procedures, reducing the overall physical and emotional burden of treatment, which could enhance the patients’ satisfaction. The cosmetic outcomes are often better, as the method can avoid unnecessarily large surgical defects, with less noticeable scarring and better functional outcomes. This method can also lower healthcare costs by minimizing the need for multiple surgeries and the associated postoperative care. Efficient use of healthcare resources, including surgical theaters and pathology services, is enhanced by reducing the number of surgeries per patient. This can free up resources for other patients and reduce the overall waiting times. While long-term savings are likely, the initial setup for the one-stop shop method may involve costs related to training, equipment, and workflow reorganization. Moreover, access to this advanced method may be limited in rural or underserved areas where specialized services are not readily available. Furthermore, preoperative margin mapping enhances the surgeon’s ability to achieve clear margins in the initial excision. Studies have shown that preoperative mapping reduces the rate of positive margins, thus decreasing the likelihood of recurrence and the need for additional surgeries [45]. In principle, all patients with BCC are eligible for the OSS-M, as they have already been informed about the procedure at a previous appointment and have already discontinued certain medications for the planned day of surgery. However, in the case of very large lesions or in areas that are difficult to access, such as the inner corner of the eye or nasal slope, the OSS-M poses a challenge in terms of both time and practicality. Accurate prediction of the excision time, efficient scheduling, effective multidisciplinary coordination, managing patient expectations, and addressing resource and infrastructure challenges are key components of the implementation. Efficient scheduling with staggered appointments and buffer time is critical to avoid delays and ensure smooth patient flow. Implementing integrated electronic health records systems can facilitate real-time information sharing and coordination among the multidisciplinary team. Moreover, developing standardized protocols and providing training for all the involved healthcare professionals can help streamline processes and reduce variability in practice.

To achieve this, a precise method of preoperative margin marking is necessary. Currently, a BCC is usually diagnosed clinically by dermoscopy and sometimes confirmed histologically by a sample biopsy, after which it is usually surgically removed with a safety margin in clinically healthy skin [3,5]. Several devices have already been tested using different methods for in vivo margin marking of BCC. In many of these studies, the dermatoscope was generally found to be advantageous over the naked eye, especially when it comes to the complete excision of the lateral margins, although there was no consensus in the literature regarding the reduction of the surgical steps [52,53,55,56,60]. However, it should be noted that most of the studies were performed on pigmented BCCs, especially when attempting to choose smaller safety margins to achieve complete excision [54,57]. Yuki et al. also pointed out that special care should be taken in less pigmented subtypes, as the dermoscope has lower sensitivity in these [51]. Due to the different distribution of the frequency of non-pigmented and pigmented subtypes in different ethnic groups, it must be considered whether the dermatoscope is a suitable tool for margin marking [105,107,108]. Nevertheless, it must be mentioned that the dermatoscope is a comparatively inexpensive method of marking the tumor margin compared to the naked eye, especially for pigmented BCC, as pigmented BCC can also be detected at smaller stages [93]. The dermatoscope is less suitable for identifying histological subtypes, as it only shows superficial structures, which is why it still recognizes superficial and nodular subtypes well but aggressive subtypes poorly [50].

Another option is OCT, which performs better than the dermatoscope in terms of margin marking, as it can still detect tumor structures under the skin that are not visible to the dermatoscope with a penetration depth of up to 1 mm [13,72]. For this reason, some studies have also examined the use of OCT for margin marking by extending the margins with OCT after dermoscopy or by subsequently comparing the defect size with the previously recorded OCT margins [26,72]. The recently published study by Akella et al. was able to show that OCT could save steps in surgery or even prevent surgery altogether, as the BCC examined here were biopsied beforehand, which can sometimes lead to complete regression due to the body’s own inflammatory response [27]. They also mentioned that nodular BCCs in particular are easy to visualize due to the easily recognizable hyporeflective ovoid structures [27]. Other subtypes, however, limit the quality of the measurement due to crust formation, and thus nests are overlooked, which leaves the question open whether the device is suitable for every subtype [27].

RCM also appears to be superior to dermoscopy for in vivo margin marking, which may be particularly advantageous for non-pigmented BCC [32]. Even the use of RCM by beginners to delineate BCC is more precise than using only the dermatoscope, although caution is advised as previous treatment with subsequent scarring may affect the outcome of the RCM measurement [34]. Also, when marking with RCM is more precise, the skin must still be incised beforehand for marking, as other markings are not visible during measurement [33]. Furthermore, the measurement also takes longer depending on the size of the lesion, as only a certain part can be measured at the same time, depending on the device [33]. Nevertheless, in vivo RCM measurement after biopsies can be useful when no BCC is clinically visible in order to make a treatment decision as to whether further surgery is necessary at all, although the low penetration depth is a limitation and has also resulted in deep-seated remnants being missed [77]. However, RCM may be more useful than dermoscopy in differentiating difficult BCC, as it detects more differences between pigmented and non-pigmented BCCs [109]. Even though the diagnostic accuracy of in vivo RCM and punch biopsy is comparably good in experienced users, subtyping is not yet completely reliable [30]. Therefore, the experience of RCM users seems to play a major role, both in vivo and especially ex vivo, which can be a major disadvantage in the decision to use this device, because intense training is required in advance (Table 2) [39,59]. For this reason, training concepts have already been tested to increase the diagnostic accuracy and enable rapid ex vivo verification of the margins [79]. Another advantage of the ex vivo RCM measurement for the OSS-M is the improvement of the contrasts in the image due to staining with acridine orange and acetic acid [15,40,79]. This facilitates the evaluation, and the possibility of digital H&E staining, which provide dermatopathologists and dermatologic surgeons with an advantage in the diagnosis as they can learn this method more easily [15,40,79]. The use of ex vivo RCM measurement directly after excision as an alternative for frozen sections can also accelerate the patient´s waiting time and thus the final wound closure and enable same-day discharge [67]. Altogether, this can have a positive impact on the patient and overall hospital resources [67].

Many authors also refer to the combination of the two devices, OCT and RCM, as RCM offers a better resolution and OCT recognizes deeper structures, which should also help to detect deeper BCC more easily [61,68]. A more reliable identification of infiltrative parts could also avoid biopsies in future [110]. RCM-OCT also shows promising results for lesions that appear inconspicuous with the dermatoscope and could also increase the specificity for the less pigmented lesions in the facial area [111]. Initial studies on the margin marking and depth determination of BCC with RCM-OCT also showed good results, as both methods complement each other well [62,63]. However, the specificity of 23.1% is again poorer when BCCs were previously biopsied, presumably due to fibrosis, although the sensitivity was 100% for biopsied and non-biopsied BCCs and thus represents an improvement compared to the individual use of the RCM [64]. For both devices, it is also possible to apply or inject various substances onto the lesion in order to enhance the contrast and thus possibly facilitate the assessment of the images [112]. Ex vivo measurement of the margins and the depth may also be a possibility to perform the surgery in one step if the device can be used perioperatively. One possibility for this would again be the combination of OCT and RCM in one device, which would make it possible to measure the tissue ex vivo in order to control the lateral margins with the RCM technique and to check the depth of the BCC with the OCT technique [65]. The results were compared with those of histology and showed that the visualization of BCCs was possible except for deep BCCs [65]. The reason for this is that compromises had to be made in the manufacture of the device to achieve a high resolution for RCM and a good depth for OCT at the expense of the penetration depth of the OCT [65].

High-frequency ultrasound (HFUS) should not be forgotten, which can also provide information on deep margins due to its high penetration depth [49]. Therefore, it is superior to the previous techniques in this respect, although there is significantly less literature on this method in margin marking than on the better-known devices [49]. Other studies also repeatedly point out the advantage of assessing deep structures, but HFUS seems to have a disadvantage compared to RCM and OCT, as the solar elastosis of the skin can make it difficult to differentiate between healthy tissue and tumor [6,113]. This issue is of course a very important factor, as most BCC are found on sun-damaged skin [6,113]. However, it must be said that ex vivo use is possible without further technical aids, as has already been demonstrated in a study by Pasquali et al., and therefore one device can be used for both measurements [46].

One of the latest developments in dermatological imaging is probably LC-OCT, combining the techniques of RCM and OCT in one device [16]. This device was also able to prove its superiority over dermoscopy alone and thus increase the diagnostic accuracy in cases of unclear BCC [42]. When differentiating between the various subtypes of BCC, a 90.4% agreement with the histology has already been described [43]. The device is a promising imaging method for dermatology, which appears to be particularly suitable for BCC but still requires further research, especially in the context of preoperative margin marking [84]. Nevertheless, a recently published case report was able to show that in vivo margin marking with LC-OCT is possible, and in this case, for an infiltrative BCC, this method was able to save skin as well as surgical steps [44]. Another recent study also demonstrated the reduction of the Mohs steps by looking at the margins of high-risk BCC using this device [45]. LC-OCT therefore seems to be a good compromise between RCM and OCT. Even if it does not have the penetration depth of OCT or a slightly poorer resolution than RCM, it can visualize the structures of the skin well and can be performed intuitively and quickly on the patient with the accompanying software [58].

For the other methods mentioned above, there is not as much literature as for the devices listed in the discussion, so no good conclusion can be drawn about their usefulness. Hyperspectral imaging system showed better performance in margin marking than clinical marking alone, but it also overestimated or underestimated the BCC sizes [97]. Moehrle et al. also mention that in the future, rapid lump examination should tell the surgeon directly whether the tumor margins are clear or not, which could lead to direct re-excision and is also a faster and easier method than RCM [100]. Although confocal laser endomicroscopy proved to be superior to magnifying glasses, it still had a high re-excision rate (27%) and is therefore inferior to the other devices [103]. The fluorescence diagnosis showed good results for non-pigmented BCC; however, the preparation of this measurement with the 6 h exposure time of the photosensitizer is rather limited for everyday clinical practice and, as mentioned, these results would first have to be tested on other ethnic groups, as this was a study from Korea [105].

In general, it should be mentioned here that the use of artificial intelligence could be helpful for the detection and subtyping of BCC, as a systematic review had already mentioned for dermoscopy, OCT and RCM [114].

## 4. Conclusions

In conclusion, there are several techniques in dermatologic imaging that can be considered for margin marking as part of an OSS method. However, so far, no device seems to fulfill all the criteria to establish such a reliable method. This means that the gold standard histology after surgery still needs to be performed. Nevertheless, many devices have shown good outcomes, resulting in reduced surgical steps for many patients, if the principles of the OSS method are used, although not all patients could profit of the OSS-M now due to the mentioned technical, practical and time limitations. The choice of the device is therefore still the personal decision of the examiner, depending on his or her preferences, but above all, on the choice between RCM, OCT, and LC-OCT (Table 2).

## 5. Future Directions

LC-OCT seems to be a promising method for this concept, so more studies should evaluate margin marking by this device. Furthermore, there should be further research on the suitability of the devices for various subtypes and pigmented and non-pigmented BCC in all ethnic groups to find out whether one device is better or worse suited for different skin types. Further studies should identify the characteristics of patients who are suitable for OSS-M (e.g., age, lesion size, localization), as well as the integration of this method into everyday clinical practice. In addition, there is still a gap concerning the reliable determination of the BCC depth in vivo, as most devices do not reach the required depth or the quality is then too poor. Thus, hopefully due to technical improvement of the penetration depth or image processing, this challenge will be overcome. Moreover, further studies on artificial intelligence in dermatological imaging should be conducted, not only to help with an easier detection and subtyping of BCC but also as a supportive possibility to examine the tumor margins for remnants and thus increase the diagnostic accuracy for experienced but above all inexperienced users. Ultimately, there should be more studies that test these devices in the context of an OSS-M to show whether this method is clinically feasible and shows the desired improvements that have been indicated in the few existing studies.

## Figures and Tables

**Table 1:** Overview of the main different devices [13,14,15,16,17,18,19,20,21,22,23,24].

Device	Method	Image Size/Penetration Depth	Resolution	Medium	Device Costs
VivoSight Dx^TM^ (Michelson Diagnostics Ltd., Maidstone, Kent, UK)	OCT	6 mm × 6 mm/1.5 mm	<10 µm	No medium necessary, plastic spacer	$$ *
deepLive^TM^ (DAMAE Medical, Paris, France)	LC-OCT	Vertical: 1.2 mm × 0.4 mm Horizontal: 1.2 mm × 0.5 mm 3D Block: 1.2 mm × 0.5 mm × 0.5 mm	Up to 1.3 µm	Paraffin oil	$$$
VivaScope^®^ 1500 (VivaScope GmbH, Munich, Germany)	RCM/in vivo	8 mm × 8 mm/ 0.3 mm	Vertical: <5.0 µm Horizontal: <1.25 µm	Ultrasound gel + paraffin oil	$$–$$$
VivaScope^®^ 3000 (VivaScope GmbH, Munich, Germany)	RCM/in vivo	Unlimited/0.3 mm	Vertical: <5.0 µm Horizontal: <1.25 µm	Ultrasound gel	$$–$$$
VivaScope^®^ 2500M-G4 (VivaScope GmbH, Munich, Germany)	RCM/ex vivo	25 mm × 25 mm/ 0.2 mm	Vertical: <5.0 µm Horizontal: <1.25 µm (taken picture: 550× magnification possible)	Ultrasound gel on the laser	$$$$
DUB^®^cutis (tpm taberna pro medicum GmbH, Lüneburg, Germany)	HFUS	12.8 mm linear/8 mm	Axial: 72 µm with 22 MHz	Ultrasound gel	$$
For example: Illuco IDS-1100 (DermoScan GmbH, Regensburg, Germany), Heine Deltaone Dermatoskop (HEINE Optotechnik GmbH & Co. KG, Gilching, Germany), DermLite^®^ DL5 (DermLite LLC, Aliso Viejo, CA, USA)	Dermatoscope (with or without polarization)	Diameter: 25 mm	Magnification: ×10	Disinfectant spray, oil or ultrasound gel	$

* $ (0–10,000 €), $$ (10,000–90,000 €), $$$ (90,000–190,000 €), $$$$ (>190,000 €).

**Table 2:** Overview of the advantages and disadvantages of the different devices [10,13,14,15,16,17,18,19,20,21,22,23,24,25,26,27,28,29,30,31,32,33,34,35,36,37,38,39,40,41,42,43,44,45,46,47,48,49,50,51,52,53,54,55,56,57,58,59,60,61,62,63,64,65,66,67,68].

Method	Role of Experience	Penetration Depth	Resolution	Subtyping	Margin Marking (Lateral Margins)	Margin Marking (Deep Margins)
OCT	+++	+++	++	+++	++	+
LC-OCT	++	++	+++	+++	++++	-
RCM/in vivo	++++	+	++++	+++	+++	-
RCM/ex vivo	++++	+	++++	+++	+++	+++
HFUS	++	++++	+	+	+	++++
Dermatoscope	++	(+)	(+)	++	+–+++ *	- ***
RCM-OCT/in vivo **	+++–++++	+–+++	++++–++	+++	+++	-
RCM-OCT/ex vivo **	+++–++++	+–+++	++++–++	+++	+++	++

* depending on the pigmentation; ** as it involves the simultaneous use of both methods, the parameters can switch; *** not possible; - none, + little/low, ++ more/better/deeper/higher, +++ a lot more/better/deeper/higher, ++++ much more/better/deeper/higher.

## Data Availability

No new data were created or analyzed in this review. Data sharing is not applicable to this article.
